# Phage Amplification Assay for Detection of Mycobacterial Infection: A Review

**DOI:** 10.3390/microorganisms9020237

**Published:** 2021-01-23

**Authors:** Monika Beinhauerova, Iva Slana

**Affiliations:** 1 Department of Microbiology and Antimicrobial Resistance, Veterinary Research Institute, Hudcova 70, 62100 Brno, Czech Republic; slana@vri.cz; 2 Department of Experimental Biology, Faculty of Science, Masaryk University, Kamenice 5, 62500 Brno, Czech Republic

**Keywords:** *Mycobacterium*, paratuberculosis, *Mycobacterium avium* subsp. *paratuberculosis*, tuberculosis, phage amplification assay, detection, viable cells

## Abstract

An important prerequisite for the effective control, timely diagnosis, and successful treatment of mycobacterial infections in both humans and animals is a rapid, specific, and sensitive detection technique. Culture is still considered the gold standard in the detection of viable mycobacteria; however, mycobacteria are extremely fastidious and slow-growing microorganisms, and therefore cultivation requires a very long incubation period to obtain results. Polymerase Chain Reaction (PCR) methods are also frequently used in the diagnosis of mycobacterial infections, providing faster and more accurate results, but are unable to distinguish between a viable and non-viable microorganism, which results in an inability to determine the success of tuberculosis patient treatment or to differentiate between an active and passive infection of animals. One suitable technique that overcomes these shortcomings mentioned is the phage amplification assay (PA). PA specifically detects viable mycobacteria present in a sample within 48 h using a lytic bacteriophage isolated from the environment. Nowadays, an alternative approach to PA, a commercial kit called Actiphage™, is also employed, providing the result within 6–8 h. In this approach, the bacteriophage is used to lyse mycobacterial cells present in the sample, and the released DNA is subsequently detected by PCR. The objective of this review is to summarize information based on the PA used for detection of mycobacteria significant in both human and veterinary medicine from various kinds of matrices.

## 1. Introduction

Mycobacteria are slow-growing, acid-fast microorganisms and highly resistant to disinfectants and other anti-microbial compounds. Many are obligate or facultative pathogens causing serious diseases in both animals and humans. *Mycobacterium avium* subsp. *paratuberculosis* (MAP) is the etiological agent of paratuberculosis or Johne’s disease, manifested as a chronic and fatal inflammatory bowel disease in domestic and wild ruminants. The economic losses of breeders caused by reduced fertility and a decrease in milk production in a paratuberculosis-infected herd are considerable. The shedding of MAP in feces and milk contributes to environmental contamination, where MAP cells can survive for several weeks or months and can be the source of further infection [1]. The disease is widespread worldwide, and the herd prevalence exceeds 50% in many European countries (Denmark, France and Italy) [2].

Other clinically significant mycobacteria include *Mycobacterium tuberculosis* complex members that cause various forms of tuberculosis (TB) in both humans and animals. From a veterinary point of view, *M. bovis* that causes bovine tuberculosis is of great importance. The disease mainly affects cattle but can also occur in humans, as well as other domestic and wild mammals [3]. Regarding human medicine, TB is still considered a leading public health problem. It is stated by the World Health Organization (WHO) that approximately one-quarter of the world’s population is infected with *Mycobacterium tuberculosis* (MT) and about ten million new tuberculosis cases occur each year, resulting in 1.2 million deaths per year. The overwhelming majority of infectious TB cases are concentrated in developing countries, with smaller shares in the Americas (2.9%) and Europe (2.5%) [4,5]. Moreover, it is estimated that annually, up to 54% of newly infected individuals remain undiagnosed [6].

Therefore, there is an urgent demand for a simple, rapid, cost-effective, and accurate diagnostic technique enabling the effective control of mycobacterial infections. However, the detection of mycobacteria in clinical, food, and environmental samples typically relies on conventional culture-dependent methods, which are slow and laboratory-intensive, but they still remain a gold standard in diagnostics [7,8]. The time required for growth of MT on solid culture media is around eight weeks, for MAP up to 16 weeks, and in the majority of cases, a decontamination step prior to cultivation is necessary [9]. This usually has an adverse effect on the viability of mycobacteria and leads to decreased sensitivity. Another option is the use of automated or semi-automated liquid culture systems, which provide reduced times for the detection of mycobacteria (about two weeks for MT). The most commonly used are BACTEC or Mycobacteria Growth Indicator Tube (MGIT) culture systems [10,11].

Conventional TB diagnosis also includes sputum smear microscopy, especially in developing countries, which is differentiated, according to the type of stain used, into fluorescence and acid-fast smear microscopy. This method is simple, rapid, and specific (97.4%); however, it suffers from low sensitivity (63.4%), at least 10^4^ cells/mL of sputum are required, and the method can also give unreliable results in HIV-infected populations [12]. According to WHO estimates, less than 20% of annual TB cases are identified as smear-positive, and although smear-negative patients are considered to be less infectious than those who are positive, they can also transmit the disease [13].

The effort to achieve faster and more accurate results has led to the introduction of molecular methods based on polymerase chain reaction (PCR), which are able to detect target mycobacteria through amplification of a specific sequence of their DNA (e.g., *F57* and IS*900* for MAP; IS*6110* for MT complex) [14,15]. The application of PCR is relatively common in MAP but not TB detection, especially due to limited resources in developing countries, which are characterized by a high burden of TB. The detection limit of PCR is typically 10^2^ CFU/mL or lower without pre-enrichment and 10^1^ CFU/mL with sample enrichment and differs depending on the type of matrix [16]. The inability to distinguish between a viable and non-viable microorganism and the susceptibility to the presence of inhibitory substances, leading to the inhibition of the PCR reaction, are major disadvantages of this approach. With regard to a possible decrease in PCR sensitivity due to the presence of inhibitors (e.g., in fecal samples), the extraction methods are an essential step in MAP detection [17].

Apart from direct detection of the infectious agent, tests targeting the host immune response are also widely employed. One of the immunological assays used for the identification of both MAP and MT is the enzyme-linked immunosorbent assay (ELISA). The limit of ELISA when determining MAP is generally reduced sensitivity, especially during the subclinical phase of the infection, and the inability to use the ELISA test for animals under 18 months of age [18,19]. The Interferon-Gamma Release Assay (IGRA) is another immune response detection method, which can aid in diagnosing MAP and MT infections. However, IGRA does not help differentiate latent TB infection from active TB. Moreover, in immuno-diagnostic assays, a cross-reaction by environmental mycobacteria may lead to the production of false positive results [20].

Besides these various alternative diagnostic methods being evaluated, tests based on bacteriophages and their natural ability to adsorb, infect, and replicate fast and specifically only within their target host cells have also shown promise in the detection of mycobacteria [6]. Bacteriophage-based tests can utilize either genetically modified reporter phages or phages in their natural state. The second approach, called the phage amplification assay (PA), exploits the natural phage infection cycle that ends with the release of progeny phages, resulting in plaque formation. While from the beginning the first approach was preferred in intensive investigations, PA is currently being paid greater attention mainly for its lower cost, simplicity, security, and no requirement for special equipment. The objective of this review is to summarize current information on PA used for the detection of mycobacteria from different matrices. The use of bacteriophages to assess the drug resistance of mycobacteria has been summarized by several studies [21,22,23], and is therefore not the subject of this review.

## 2. History and Principle of Phage Amplification Assay

The interest in mycobacteriophages, i.e., viruses that infect mycobacterial hosts, as an important research tool in diagnosis and drug susceptibility testing of *Mycobacterium* species has renewed since their first identification over 70 years ago [24], mainly due to progress in the understanding of their structure and function [14]. The idea of using bacteriophages for bacterial target detection by PA came from observation of the effects of anti-TB drugs on mycobacteriophages in which streptomycin prevented phage replication in *Mycobacterium smegmatis,* but not in resistant strains [25]. However, the first application of the PA method is dated to 1983, when Hirsh and Martin determined the presence of *Salmonella* spp. in pure culture [26] and milk samples using *Salmonella*-specific phage Felix-O1 [27]. The increase in bacteriophage concentration as a result of successful infection was detected by high-performance liquid chromatography (HPLC). Nevertheless, costs and complexities prevented further use of HPLC in connection with PA, instead, many other end-point analyses have been explored, such as fluorochromic staining [28], real-time PCR [29] or matrix-assisted laser desorption/ionization mass spectrometry [30].

Early studies also focused on the use of reporter phages (e.g., TM4, T4, phAE142) with genetically engineered marker genes to produce an easy-to-measure visual signal allowing the host cell to be identified when marker genes were expressed inside the host cell. These signals include colorimetric signals, bioluminescence and fluorescence yielded by β-galactosidase, bacterial or firefly luciferase, and green fluorescent protein, respectively [31,32,33,34]. However, this approach was costly and often suffered from legal and prohibitive regulatory issues due to the recombinant nature of the bacteriophages. Thus, the PA method described by McNerney et al. [35] using native lytic mycobacteriophage D29, isolated from soil by Froman et al. [36], became preferable in mycobacteria detection and is currently still in clinical use.

**Figure 1:**
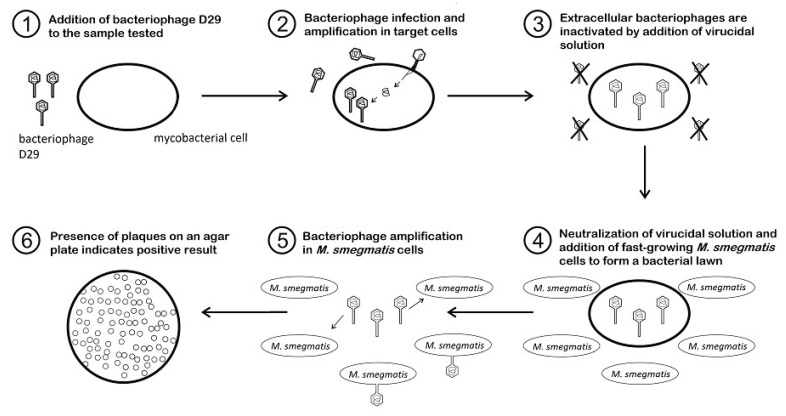
The schematic representation of the phage amplification assay methodology.

**Figure 2:**
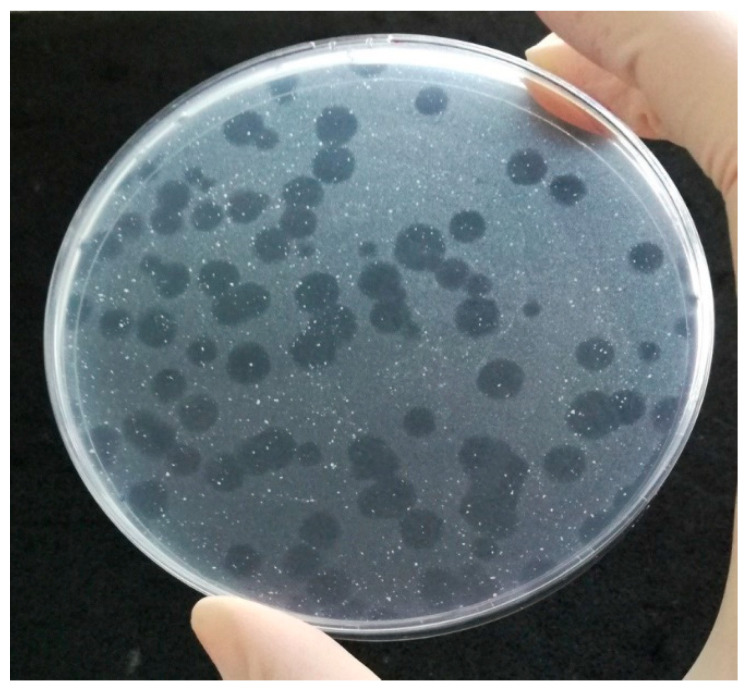
The positive sample determined by phage amplification assay.

The PA method provides information on the presence of viable mycobacterial cells (as bacteriophages are capable of replicating only within viable cells) within 24 to 48 h, what makes it far more sensitive than conventional cultivation techniques (<100 CFU/mL) and no complicated instruments are required [37]. Different kinds of matrices, such as milk and milk products, feces, sputum, blood, or tissue can be tested by PA. Some types of matrices must be decontaminated first (e.g., feces, sputum, or urine) to reduce most bacteria of contaminant microflora. Subsequently, the target mycobacterial cells present in the specimen are infected by the bacteriophage D29 added to the specimen (Figure 1). In the next step, all exogenous bacteriophages that have not infected host cells are inactivated by the virucidal solution. Only bacteriophages that penetrated host cells and are therefore protected within viable mycobacteria can survive this treatment. The virucide is neutralized and a population of non-pathogenic, fast growing helper cells of *M. smegmatis*, also susceptible to phage infection, are added. In the meantime, the surviving bacteriophages continue to replicate until numbers of progeny phages are released into the culture medium due to cell lysis. Progeny bacteriophages repeatedly infect helper cells, replicate inside them, and finally lyse them, which can be seen after overnight incubation as clear areas (plaques) in the lawn of confluent growth of *M. smegmatis* (Figure 2). Each plaque may derive from a single viable mycobacterial cell or a clump of these cells in the original specimen. If no viable target bacterium is present in the original sample, there will be no phage replication and therefore no phages to detect as plaques in a lawn of *M. smegmatis* [14].

With the ability to use fast-growing *M. smegmatis*, instead of target cells, for the creation of a background bacterial lawn allowing plaques to be counted on a plate, the time required to obtain results is significantly reduced. If a high number of mycobacterial cells are present in a specimen (>300 PFU/mL), the lawn of *M. smegmatis* is completely lysed, and therefore for acquisition of quantitative results for PA, it is necessary to dilute samples before plating [38].

Bacteriophage D29 is a Cluster A2 mycobacteriophage with only a lytic pathway of infecting hosts, belonging to the *Caudovirales* order of viruses. Like most mycobacteriophages (>90%), D29 has siphoviral morphology with long flexible tails [39]. The D29 genome is composed of a linear double-stranded DNA with a length of 49,136 base pairs and a GC content of 63.6% [39,40,41]. Bacteriophage D29 is one of the mycobacteriophages with a broad range of *Mycobacterium* sp. hosts, indicating the ability to bind to receptors that are common among many mycobacterial species. The broad range of bacteriophage D29 hosts is essential for PA detection, where both slow-growing and fast-growing mycobacteria need to be infected. The D29 host range is to some extent similar to that of the mycobacteriophages Bxz2, L5, and TM4. Productive infection of bacteriophage D29 was observed for *M. smegmatis*, *M. tuberculosis*, *M. bovis* BCG, *M. kansasii*, *M. gastri*, and *M. ulcerans*, but not for *M. marinum*, *M. fortuitum*, and *M. chelonae* strains [5,42]. Inconsistent results were achieved for *M. avium* complex members. In a study by Rybniker et al. [42], bacteriophage D29 proved to be lytic in all three strains of *M. avium*; however, plaque formation was not observed in avian strains of *M. avium* tested by Froman et al. [36]. Similarly, only one of two strains of *M. scrofulaceum* allowed D29 growth, suggesting that not all mycobacterial strains are susceptible to phage infection and this phenomenon should be kept in mind when using PA for the detection of mycobacteria [42]. On the other hand, the study by Foddai and Grant [43] showed susceptibility of all 43 MAP strains to D29 infection, demonstrating the suitability of PA utilization for MAP detection. Besides mycobacteriophages with a broad range of mycobacterial hosts, those that are able to propagate in only one to four mycobacterial species have been described (Che8, Cooper, Wildcat) [42].

Formerly, the commercial phage-based kit named *FASTPlaque*TB™ (FPTB) and its variant PhageTek MB assay (Biotec Laboratories Ltd., Ipswich, UK) were available and primarily designed for *M. tuberculosis* detection in human sputum specimens. Now it is possible to use an in-house variant of the assay, which means a laboratory-developed PA assay not significantly different from the commercial one. This could represent a suitable alternative to PCR tests, especially in low-income countries, because it relies only on basic microbiological techniques. Biotec Laboratories Ltd. also designed variants of these tests named *FASTPlaque*TB-*RIF*, *FASTPlaque*-TB-MDRi™ and its later version *FASTPlaque*-TB-Response™, which determined drug resistance in culture isolates. This technique using phages for determination of drug resistance has been used in several studies [44,45] and reviewed by Minion and Pai [23].

Due to the fact that bacteriophage D29 has a broad host range and is able to infect various species within the genus *Mycobacterium*, in order to achieve sufficient specificity for target mycobacteria, the assay is combined with subsequent PCR, during which the target-specific amplification of DNA from mycobacterial cells detected by phage occurs [46]. This method is called plaque PCR. Nonetheless, it was also observed that PA-positive samples can give negative results by plaque IS*900* PCR [47], which may be due to the insensitivity of PCR and difficulty in detecting DNA originating from only a single MAP cell in a plaque. In this regard, a multicopy PCR target is preferable for use [46]. Another more likely explanation is that the virucidal solution does not inactivate all D29 phages added to the sample, and thus certain plaques can arise as a result of lysis of only *M. smegmatis* cells. This assumption leads to the conviction that confirmation of results by plaque PCR is essential for this method, also taking into account the possible presence of environmental mycobacteria in the sample, with plaque PCR being best performed on more than one plaque [47,48,49]. In this context, limits for the interpretation of FPTB test results have also been established. The specimen is evaluated as positive if the number of plaques is 20 or more, and as negative if 0 to 19 plaques are obtained [38]. Potential contamination is controlled on every occasion through positive and negative controls as well.

The weakness of PA can be an unsuccessful infection in a substantial proportion of bacteria present in the sample, which may range from half to four-fifths of the estimated CFU, and may be caused by a number of factors; e.g., phage replication cannot occur in bacteria that are dormant or have disrupted replication [37]. The duration of sample storage, a decontamination pre-step, and receiving of anti-TB therapy in the diseased group may influence this, and since PA can only detect viable mycobacteria and its threshold of detection is about 10–100 bacilli based on the matrix tested, any factor that impacts viability of mycobacteria may result in decreased sensitivity [6]. Study results also suggest that a sizable fraction of target cells is rendered in a nonviable state, which is caused by reactive oxygen species (ROS) that are released by host cells after phage infection and act extracellularly on other cells and kill them [50].

Recently, “conventional” PA has been modified and a commercial kit called Actiphage™ (PBD Biotech, Suffolk, UK) is now available, overcoming some of the shortcomings mentioned. The principle of this novel approach is to utilize the lysing capability of bacteriophages, resulting in the release of mycobacterial DNA, which, after lysate filtration and purification, is detected by PCR. The main advantage is the reduced time taken in achieving results (within 6–8 h) and there being no need to prepare agar or a *M. smegmatis* culture. The limit of detection (LOD) is less than 10 cells and specificity is determined by the PCR used [51].

In the following paragraphs, the use of the PA method for the detection of mycobacteria in various types of matrices is described. The paragraphs are divided according to the mycobacterial targets determined and type of method used (direct PA and PA combined with magnetic separation). Information from all articles published so far regarding PA methodology for mycobacterial detection is also summarized in the following tables.

## 3. Phage Amplification Assay for Detection of Mycobacteria Significant for Human Medicine

In human medicine, MT complex bacteria are a major cause of mycobacterial infections. Tuberculosis is the disease most prevalent in developing countries with low resources, where PA may support prompt and low-cost diagnosis as well as high sensitivity, which are prerequisites for controlling this life-threatening disease. In general, the sputum represents the most common clinical sample in TB diagnosis, and therefore studies based on PA technology focused mainly on sputum as a matrix. To a lesser extent, however, other respiratory specimens, blood or urine were also analyzed [12,52,53].

In order to evaluate and optimize the developed PA, a few experiments were performed with artificially contaminated samples (Table 1). These experiments included mycobacterial suspensions in growing media (Middlebrook 7H9) or artificially contaminated smear-negative sputum, but the vast majority of summarized studies focused on testing naturally contaminated clinical specimens and comparing PA methodology with other commonly used diagnostic techniques (culture, smear microscopy, PCR; Table 2). In addition, PA has been most widely employed for the detection of MT complex bacteria using the formerly commercially available FPTB kit, although a few studies have also dealt with in-house assays [54].

The first published information about PA optimization, based on a chemical inactivation procedure effective on free phages in solution, emerged in 1998. For testing, the indicator strain (*M. smegmatis*) was used. The advantage of using this strain, besides its overnight growth (quickly achieved results), is a low reported incidence of pathogenicity [35]. All publications in Table 1 describe the evaluation of the method in connection with practical usage. The most tested mycobacteriophage is D29 connected with *M. smegmatis* application as a model non-pathogenic and *M. tuberculosis* as a pathogenic mycobacterial strain.

Optimization of the PA-based method for the detection of MT complex bacteria directly from the sputum was first described by Park et al. [55]. The optimal infection time prior to the addition of virucide was found to be between one and three hours with an extension of the plating time leading to a several-fold increase in signal (corresponding to the burst size). However, the study by McNerney et al. [37] showed that delaying plating until after lysis will not improve LOD. The relatively expensive component oleate-albumin-dextrose-catalase (OADC), which is used as a supplement of culture medium added to the sample after treatment with virucide to inactivate it, was replaced with cost-effective sodium citrate supplemented with calcium chloride, showing greater protection from virucidal solution than OADC. Nevertheless, this solution has not been adopted thus far [55]. Authors also reported an alternative to the plate-based final step, with detection of progeny phages in *M. smegmatis* liquid culture using 3-(4,5-dimethylthiazol-2-yl)-2,5-diphenyl-2*H*-tetrazolium bromide (MTT) color indicator treatment with a LOD of 60 viable MT cells in 1 mL of sputum.

In the study by McNerney et al. [37], the length of the latent period of phage D29 was found to vary among different MT strains, ranging from 2 to 5 h post-inoculation. According to the authors, the presence of aggregates of bacteria can affect the kinetics of infection. Specimens containing 100 CFU of *M. bovis* BCG/mL of culture medium were repeatedly found to be positive, showing a sensitivity 100-fold greater than that of smear microscopy. The best efficiency of D29 infection was observed when a concentration of phages of 8 × 10^7^ PFU/mL was used, and the number of plaques decreased with inoculates exceeding 10^8^ PFU/mL, suggesting that abortive infection could occur at higher concentrations.

### Detection of M. tuberculosis Complex Members in Human Clinical Samples by Phage Amplification Assay

Initial clinical studies of phage-based assays on human clinical samples were conducted in 2002 and since then, many studies have investigated the performance characteristics of the commercial FPTB technique or in-house PA for the direct detection of MT (Table 2); predominantly in developing countries with a high burden of TB (South Africa, India, Pakistan). The sputum smear microscopy represents, due to its simplicity and low cost, the most commonly used and often also the only laboratory tool in diagnostics there. However, because of the low sensitivity of smear microscopy, it is assumed that a significant number of TB cases are likely to be missed [12]. Therefore, there is an urgent need for an alternative sensitive method, which should also provide fast results.

The reported performance data of the commercial phage-based kit varies across different publications. The results of nine studies show that FPTB tests have a high specificity (range: 77.5–98.7%) but modest and highly variable sensitivity (range: 20.7–90.7%) with sputum samples, compared to culture. Besides differences in sample storage duration, sample processing, and extent of patients receiving anti-TB therapy, the proportion of smear-positive and smear-negative cases may reflect this heterogeneity in the FPTB sensitivity values determined. Muzaffar et al. [13] reported that in smear-positive specimens, the sensitivity of FPTB tests reaches up to 87.4%, with a specificity of 88.2%. In contrast, the sensitivity for smear-negative specimens was 67.1% with a specificity of 98.4%, which can be explained by the threshold value of detection of PA that was assessed to be about 100 viable MT bacilli/mL. The impact of bacillary load on the PA performance was also observed by Prakash et al. [8] when the sensitivity decreased at a very low bacillary load (smear grade 1 and scanty results). The positive (PPV) and negative (NPV) predictive values of PA also showed significant heterogeneity, especially when a FPTB kit was used for detection of MT in sputum. With culture used as a reference method, the PPV and NPV of FPTB ranged from 32% to 97.5% and from 64.6% to 96%, respectively; but when samples other than sputum were included in the analysis, both PPV and NPV estimates exceeded 80%. It is known that the administration of anti-TB treatment to patients prior to collecting specimens leads to a reduction in the number of viable MT cells in the specimen, which therefore cannot be detected by PA; however, from the summarized data (Table 2) it is not clear if PA achieves higher sensitivity values in samples from patients not treated before sampling. This issue should be further examined. It is also important to consider that laboratory staff experiences or laboratory facilities may influence the level of FPTB performance, underlining the need for adequate training in FPTB use. Nevertheless, the overall performance of FPTB is significantly better than that of smear microscopy, which might suggest a very effective role for PA in early diagnosis of both smear-positive and smear-negative patients with low numbers of viable mycobacteria in specimens.

When FPTB was used for the detection of MT complex bacteria in urine specimens, the sensitivity, specificity, and overall accuracy of FPTB were 100% each, compared to culture [14]. According to the authors, the reason for such a high sensitivity of the method for urine samples may be a larger sample volume used (45 mL) in comparison to sputum specimens, or the decrease in viscosity of urine versus sputum. In addition, the performance parameters of FPTB were superior to that of IS*6110* PCR, whose sensitivity, specificity, and overall accuracy values were 67%, 100%, and 91%, respectively, compared to culture. However, only 22 urine samples were analyzed, and therefore verification of results with a larger number of specimens is needed.

As indicated above, the group of sputum-negative paucibacillary patients represents a significant challenge for the determination of a definitive diagnosis. Biswas et al. [59] evaluated the accuracy of PA, focusing on this diagnostically difficult group of patients. Generally, cultivation serves as the major comparative method to assess the accuracy of PA. However, in this study, the TB-positive patients were determined on the basis of clinical features with regard to the paucibacillary nature of TB. Although the performance of culture reaching 70% sensitivity was superior to that of FPTB (58.8% sensitivity), 18.52% of the culture-negative cases were found to be FPTB-positive, which may indicate that culture is not the most appropriate reference standard investigating PA performance due to its moderate sensitivity. In addition, Zhu et al. [63] reported that the overall specificity of PA was 71.6% when compared with culture, whilst when using a patient’s clinical features as the reference standard, specificity was 99%.

A study by Bonnet et al. [7] is the only report dealing with the MT complex bacteria detection in smear-negative sputum samples originating from HIV-infected patients. HIV infection was documented to potentially affect the rate of viable mycobacteria present in sputum specimens [6]. Achieved values of sensitivity and specificity were 31.2% and 94.9%, respectively, compared to culture in all patients examined, and 33.3% and 93.9%, respectively, in HIV-positive patients demonstrating comparable results between these two groups evaluated. However, a comparison of FPTB performance between HIV-negative and HIV-positive patients needs to be performed, which was not possible here due to the small number of samples from HIV-negative patients.

According to FPTB kit manufacturers, before performing phage-based assays the decontamination treatment of patient specimens is required to suppress the growth of competitive microflora. Decontamination was thus carried out in all studies listed in Table 2, except for the study by Verma et al. [53], which tested blood samples. Nevertheless, it was noted that the decontamination step usually leads to a substantial decrease in final sensitivity [37]. In addition, despite decontamination, overgrowth of contaminants on an agar plate impeding interpretation of PA results can still be observed. This issue can be alleviated by the addition of an antimicrobial agent to FPTB media (e.g., Microclens), which is of no adverse effect on assay performance. However, even in this case, complete inhibition of the contaminating bacteria growth was not achieved [13]. In a study by Mbulo et al. [54], two different protocols were used to decontaminate sputum. When using the FPTB manufacturer’s decontamination protocol, a microbial contamination of 40.4% was observed; however, when the modified Petroff’s method (WHO recommended protocol) was used, only about 5% of specimens were found to be contaminated on Löwenstein–Jensen (LJ) medium. Moreover, the rapid initiation of PA is essential, as a substantially better sensitivity was achieved when samples were processed daily compared to processing of samples twice a week [8,56].

A study dealing with the comparison of performance characteristics of the FPTB kit and an in-house PA in the MT complex bacteria detection in sputum samples was published by Mbulo et al. [54]. In this study, the in-house PA proved to have similar specificity but higher sensitivity (45.3%) than the commercial FPTB kit (20.7–32%). On the other hand, many publications using the FPTB kit for MT detection in sputum showed results of accuracy to be much higher than those of the in-house PA reported in this study [8,12]. Significantly higher values of sensitivity in the MT detection in sputum by in-house PA were reached by Zhu et al. [63]. The reported sensitivity of 54.8%, acquired during comparison of PA results with the reference standard defined by clinical diagnosis, was superior to that of LJ culture (37.7%). Better estimation of FPTB sensitivity compared to LJ culture were reported also by Singh et al. [60]; however, the majority of publications describe the opposite results [58,59], making culture the more reliable method of detecting MT than PA.

In most studies, verification of PA-positive specimens for the presence of MT complex bacteria was not performed. However, the prevalence of non-tuberculosis mycobacteria infections is low in developing countries (e.g., Pakistan, Zambia) and specificity remains high [65]; however, in areas where the prevalence of these microorganisms is higher (Europe) the end-point analysis by plaque PCR is required to maintain sufficient specificity. Nevertheless, as far as resource-limited developing countries are considered, the idea of using these confirmatory tests is not realistic. In the studies listed here, though, the final results were interpreted in conjunction with another diagnostic method to avoid false positive results.

The use of a novel adaptation of PA called Actiphage™, in which mycobacterial DNA is analyzed by PCR after cell lysis caused by phage D29, was reported by Verma et al. [53]. The experiment included the detection of MT in blood samples from immunocompetent patients with active and incipient TB. The method proved to be very effective in detection of pulmonary TB, reaching 73% sensitivity and 94% specificity, which demonstrates the great potential of the method in early diagnostics of pulmonary TB. However, it remains to be seen how this method will be adopted in developing countries, in terms of the material and equipment costs for PCR.

Although PA achieves higher accuracy than microscopy, if all aspects are taken into account, such as complexity, costs, contamination rate, the requirement for an established laboratory infrastructure, and especially heterogeneity of the sensitivity values obtained, smear microscopy seems to remain the main laboratory tool in the TB diagnosis in countries with fewer resources. PA rapidity could be an advantage in comparison with culture and as PA relies on the presence of viable cells, it would be useful for monitoring response to therapy; however, PA cannot replace conventional culture at this time. Considering a certain rate of PA false negative results, it has been proposed for patients with a high clinical suspicion of TB and negative smear microscopy results to test a sputum sample using both PA and culture. This combination of methods should suit laboratories in low-resource areas, ensuring a more accurate diagnosis of TB. This would also be advantageous in cases of PA false positive smear-negative sputum, where virucide fails to eliminate all free phages [66].

## 4. Phage Amplification Assay for Detection of Mycobacteria Significant for Veterinary Medicine

Although the bacteriophage-based FPTB kit was originally targeted for the direct detection of viable MT complex bacteria from sputum specimens, after assessment of bacteriophage D29 host range and ascertainment of phage capability to replicate also in MAP or *M. bovis* cells, many studies have started to investigate the possibility of PA use for the detection of these mycobacteria in various matrices, including milk, cheese, powdered infant formula (PIF), or blood (Table 3). Before moving on to clinical specimen testing, numerous optimization experiments were performed using artificial contamination of samples to exactly evaluate PA (commercial or in-house) for the selected matrix (Table 4). The evaluation was done mainly on ultra-heat treated (UHT) milk or cultivation medium (Middlebrook, FPTB), in two cases on blood, and in one case on skim milk powder. For spiking experiments, MAP cells were the most often used. From a methodological point of view, usage of different mycobacterial species (mostly rapidly growing species) is understandable and, due to the same composition of bacterial cell wall, acceptable. From the point of view of practical usage and potential mycobacteria occurrence detection, MAP or *M. bovis* predominate. The dose of mycobacterial cells used for artificial contamination ranged from 10^0^ to 10^7^ CFU/mL or PFU/mL of milk and culture medium specimens, 10^0^ to 10^3^ CFU/g of skim milk powder, and 10^0^ to 10^4^ PFU/mL of blood. *M. smegmatis* cells were used as a fast-growing model organism for MAP to investigate environmental factors affecting phage infection [67], or as a negative control verifying specificity of IS*900* plaque PCR [46] and for burst time determination [68]. *M. tuberculosis* and *M. bovis* BCG were used to develop a multiplex PCR to allow simultaneous amplification of either MAP or MT complex–member specific sequences from plaque specimens [46]. Otherwise, *M. bovis* BCG is usually used as a surrogate for the containment level 3 pathogen *M. bovis*.

Stanley et al. [46] were the first to describe successful FPTB assay application for the detection of MAP cells. For molecular confirmation of cells detected by PA and the specificity addition to the assay, end-point PCR was introduced during which amplification of specific sequences (IS*900*, IS*6110* and IS*1081*) of mycobacterial DNA from plaques occurs. This step is necessary due to the possible presence of environmental mycobacteria in the sample. Moreover, the experiment with heat-killed MAP cells was conducted, proving that only viable cells can be detected by the FPTB assay.

The more accurate enumeration of viable MAP cells in the original sample is possible due to the optimization of PA conditions by Foddai et al. [68]. Close to 100% correlation between PFU/mL and CFU/mL counts of MAP was achieved by an optimized procedure, with the mean differences between PFU/mL and CFU/mL being 0.45 log_10_ and 0.23 log_10_ for spiked medium and UHT milk, respectively. The optimized PA achieves a detection limit of 6.2 × 10^0^ PFU/mL, 1.7 × 10^1^ PFU/mL, and 8.7 × 10^0^ PFU/mL of spiked medium, UHT milk, and raw milk, respectively [74], and has been shown to be useful in assessment of the viability of MAP cells subjected to physical treatment [75].

Recently, Swift et al. [49] investigated the use of PA methodology for *M. bovis* BCG detection in blood. Since it was found earlier [76] that mycobacteria cells present in circulating blood are located within white blood cells, the peripheral blood mononuclear cells fraction (PBMC) was isolated following the spiking of heparinized sheep blood by the cell suspension, and after lysis of PBMC, the released mycobacteria were analyzed by PA. The cell suspensions containing 10^5^–10^1^ *M. bovis* BCG cells, used to inoculate sheep blood, yielded plaques; however, the countable amount of plaques (65 ± 13) was reached when 10^2^ *M. bovis* BCG cells were added with an efficiency of the *M. bovis* BCG cells uptake of at least 50%. The identity of detected mycobacterial cells was determined by PCR and recombinase polymerase amplification (RPA) following DNA extraction from individual plaques using agarose gel-DNA extraction columns. Both methods were capable of detecting the IS*6110* genetic element in the sample composed of DNA extracted from three to five plaques; however, only the RPA method was able to consistently detect the IS*6110* element when only one or two plaques were present in the sample, probably due to its lower susceptibility to inhibition by blood components [77]. The established LOD of the PA-RPA method was about 10 viable cells per sample [49].

Because PA has been shown to be very effective in detecting the mycobacteria’s presence in blood samples, the Actiphage™ kit performance was also examined on this type of matrix [51]. As in the previous study, sheep blood specimens were inoculated with diluted mycobacterial suspension and mycobacteria recovered from isolated PBMC were determined using Actiphage™ followed by PCR, with the results being compared to the original PA. The Actiphage™ kit reached a lower LOD (<10 cells) than the original PA in the detection of MAP or clinical isolate of *M. bovis* in blood, while detecting *M. bovis* BCG strain provided the same LOD for both tests. In addition, Actiphage™ has shown to be less laborious than original PA and suitable for high-throughput testing.

### Detection of M. avium subsp. paratuberculosis and M. bovis in Real Samples by Phage Amplification Assay

In the diagnosis of animals infected with paratuberculosis or bovine tuberculosis, milk represents one of the most frequently investigated matrices. Stanley et al. [46] has shown for the first time the successful application of the PA method using the FPTB kit for the viable MAP detection in milk samples originating from naturally infected animals with results available within two days. Later, the study by Botsaris et al. [69] performed in Cyprus and examining all cattle herds for the presence of MAP reported 22.2% of 225 cattle bulk tank milk (BTM) samples detected positive by FPTB. These results are in accordance with the level of prevalence obtained by IS*900* quantitative PCR (qPCR) documented by Slana et al. [78]. In contrast, only 0.9% of BTM samples tested positive with culture. This difference could be explained by the fact that the chemical decontamination of specimens is required prior to conventional culture, which leads to the reduction in detectable MAP by at least one log_10_ and thus to an increase in the detection limit of culture [79]. Since PA does not require decontamination for these animal specimens, it is far more sensitive than culture techniques. In addition, a cut-off value of 59 PFU per 50 mL BTM sample was assessed to accurately predict whether the sample contained MAP without the need for plaque PCR identification, which reached 90% sensitivity and 99% specificity. However, the cut-off value can only be applied in MT complex-free cattle populations (e.g., Cyprus) [48].

To explore whether MAP could survive the cheese-making process, 28 cheese specimens from retail outlets in Cyprus were investigated. Although the presence of MAP DNA was detected by PCR in 25% of samples, none was found to be positive for viable MAP by FPTB or culture [69]. This indicates that either only dead or dormant cells were present in specimens, or MAP cell surfaces were affected, reducing phage ability to adsorb to cells.

The association between MAP and the development of Crohn’s disease in humans remains a controversial subject; however, there is still a potential risk to human health through the consumption of food products or water contaminated with MAP [80]. In addition to the direct pathogenic effect of MAP on the host, the participation of indirect immunomodulatory impacts following exposure to MAP on the development of disease has been proposed [81]. Pasteurization is a major means of controlling the MAP transmission to food products; however, MAP has been shown to be present in retail pasteurized milk by PCR or culture, indicating the ability of MAP to survive high-temperature, short-duration pasteurization treatments [82]. Using an in-house PA-PCR for MAP detection in retail pasteurized milk samples in the UK, the prevalence of viable MAP in this type of sample was demonstrated to be higher (10%) than has been estimated so far by means of culture techniques (1.7–6.7%) [71,83]. Overall, 6.8% of samples contained one to two viable MAP cells per 50 mL, reaching a much lower LOD compared to culture or PCR [84,85]. This suggests the potential of the PA method in monitoring the efficacy of milk pasteurization processes.

Based on these findings, PIF samples were also investigated for the presence of viable MAP, as they are made from pasteurized milk [70]. The LOD of the PA method for PIF samples was determined to be approximately 10 CFU/mL of reconstituted PIF, which is lower than levels of MAP estimated to be present in naturally contaminated products based on quantification of DNA [86]. This demonstrates the suitability of PA for purposes of testing naturally contaminated PIF samples. Four out of 32 PIF samples collected from retailers in Cyprus, and originating from ten different producers, were found to contain viable MAP cells by PA-PCR, which is one more sample than determined by culture. These findings indicate that MAP can survive not only pasteurization but also further manufacturing processing of PIF, posing a risk in infants’ exposure to MAP [70].

The identification of disseminated infections and bacteremia caused by *M. bovis* in cattle remains a challenge due to the unreliability of commonly used diagnostic tools [87]. The study by Swift et al. [49] is so far the only available report on the use of conventional PA for rapid detection of MT complex bacteremia in animals. The presence of viable MT complex cells was investigated in the PBMC fraction of blood specimens originating from single comparative cervical intradermal tuberculin (SCCIT)-positive and SCCIT-negative cattle herds. The optimized PA-RPA assay revealed mycobacteremia in 66% of SCCIT-positive specimens and no MT complex cells were detected in specimens from a SCCIT-negative herd. In addition, if lesions were observed in tissue from SCCIT-positive cattle, PA gave a positive result in 85% of cases, and intriguingly, 57% of the animals without visible lesions had detectable mycobacteremia. Concurrently, significantly more MT complex bacteria (95%) were detected by the Actiphage™ kit than using PA-RPA when the same SCCIT-positive samples were analyzed [51]. All samples from animals with lesions and 93% of samples from animals without lesions tested positive by Actiphage™. Using the SCITT status as a comparator, Actiphage™ reached a sensitivity of 95% and specificity of 100%. The higher sensitivity can be explained by the fact that the sample material remains in one tube throughout the test, which results in a lower risk of sample loss.

The performance comparison of Actiphage™ to the original PA for the detection of MAP cells in cattle blood was also performed [51]. While only 40% of samples originating from experimentally infected cattle tested positive using original PA-PCR, Actiphage™ detected viable MAP in 87% of these specimens, which again suggests that Actiphage™ reaches a higher sensitivity than original PA. However, detectable levels of MAP were also identified in two blood specimens from a negative control group, and thus a question about the specificity of Actiphage™ was raised. As the resulting specificity is determined by the end-point PCR used, the IS*900* PCR was evaluated by testing 45 DNA samples prepared from a negative control herd. No positive result was recorded, suggesting that the false positive results did not arise due to the specificity of the Actiphage™ method. A remarkable result was achieved by Haas et al. [88], who were able to identify active MAP infection in newborn calves using the Actiphage™ kit with good reproducibility. Through monthly analyses of blood samples for a period of six months, transient bacteremia was identified in calves in the first days of life. The successful application of both PA methods on blood samples suggests PA is a useful tool to study *M. bovis* or MAP infections, which may allow better understanding of the course of tuberculosis and paratuberculosis in domestic and wild animals.

## 5. Combination of Phage Amplification Assay with Peptide-Mediated Magnetic Separation for Detection of Mycobacteria

In light of the broader host range of mycobacteriophage D29, an additional selective step prior to phage infection was considered necessary to introduce a higher specificity for PA. One option could be the utilization of various magnetic separation approaches, which have become routine in veterinary and food microbiology laboratories, where they serve as a common tool for detection and isolation of pathogens such as *Salmonella* spp. or *Escherichia coli* from food and veterinary samples [89,90]. The magnetic separation can selectively capture and concentrate target bacteria from a sample while removing contaminating microorganisms, as well as other potential inhibitors. The principle of the method is a selective interaction between cell surface structures of the target bacterium and specific binding ligands coated on paramagnetic beads. These ligands can be either monoclonal and polyclonal antibodies (immunomagnetic separation; IMS) or biotinylated and non-biotinylated species-specific peptides (peptide-mediated magnetic separation; PMS). Magnetic separation is basically used to improve the analytical sensitivity and specificity of the subsequent detection method, which can be culture, microscopy, an antigen detection immunoassay, PCR, or PA [91].

Numerous studies focusing on the optimization of PA in conjunction with magnetic separation on different types of matrices (culture medium, milk, feces, or blood) with artificially contaminated samples have been published and are summarized in Table 5. For the evaluation of the protocol performance, different mycobacterial species were tested. The most often used were MAP isolates with a dose of cells spiked ranging from 10^0^ to 10^6^ CFU/mL or PFU/mL of culture medium or milk and 10^0^ to 10^4^ PFU/mL of blood. Various environmental mycobacteria or non-mycobacterial raw milk isolates were used to assess the specificity of the optimized method.

Different paramagnetic beads coated with antibodies and/or peptides were evaluated with the aim of determining the capture efficiency and specificity for MAP cells [91,94]. The most common beads for MAP capture are MyOne Tosylactivated Dynabeads, which were used in all studies summarized in Table 5 and Table 6. The experiments dealing with the efficiency of the capture of MAP cells and nonspecific binding of other *Mycobacterium* spp. by different paramagnetic beads (e.g., MyOne Carboxylic acid Dynabeads, MyOne Streptavidin-T1 Dynabeads) coated with a polyclonal antibody (S624) and peptides (biotinylated or non-biotinylated aMp3 and aMptD) showed that a 50:50 mixture of MyOne Tosylactivated Dynabeads coated with biotinylated peptides aMp3 and aMptD reached the highest capture efficiency for MAP. This approach in conjunction with PA achieved a 98.5% capture efficiency of viable MAP from culture medium with 5.5% nonspecific recovery of other *Mycobacteria* spp. and has become the most common approach used in PMS-PA for the detection of MAP cells [91]. However, since the PMS step could also recover other *Mycobacterium* spp., plaque PCR is still required after PMS-PA to confirm the identity of the mycobacteria in a sample.

The mean 50% limit of detection (LOD_50_) of automated PMS-PA was assessed to be 1.9 and 7.3 PFU/mL of spiked broth and UHT milk samples, respectively [91]. With 50 mL milk samples, which represents the most common volume of milk tested for the presence of MAP in animals, the reached LOD_50_ was 0.93 PFU per 50 mL of milk, making this approach more sensitive in MAP detection than existing qPCR and conventional culture methods [43]. However, to achieve accurate viable MAP enumeration by this protocol, optimal milk sample storage and preparation had to be ensured. For the usage of PMS-PA for milk, it is crucial to know that: (i) maximal numbers of MAP cells were found to sediment in the pellet fraction upon centrifugation at 2500 *g* for 15 min at ambient temperature; (ii) milk specimens should be refrigerated at 4 °C after collection and MAP testing should commence within 24 h, or, when not possible, specimens can be frozen at −70 °C for up to one month without significant loss of MAP viability [92]. However, the PMS-PA performance characteristics obtained by Butot et al. [94] were inconsistent with those published by Foddai and Grant [43]. Using the most probable number enumeration technique to determine the reference value, the LOD_50_ of PMS-PA was assessed to be 3.7 log_10_ CFU/50 mL of both raw and heat-treated milk.

The connection of PMS-PA methodology with ELISA in a competition assay format in which released progeny phages are detected by a polyclonal antibody was also evaluated [95]. This approach eliminates the need to maintain fresh culture of *M. smegmatis* or problems with enumeration of samples containing a high number of MAP. The developed phage-based immunoassay with a dynamic range estimated to be approximately 3 × 10^2^–6 × 10^8^ phage/mL represents a rapid screening method allowing the detection of low numbers of viable MAP in milk and fecal samples, which has potential applications within the veterinary or food industries.

### Detection of M. avium subsp. paratuberculosis in Real Samples by Phage Amplification Assay in Combination with Peptide-Mediated Magnetic Separation

Evaluations of the PMS-PA connection were initiated in 2010 and the first publication on testing real samples occurred one year later (Table 6). The main reasons for using PMS were the presence of components that inhibited PA to such an extent that the sensitivity of PA was not useful, or low numbers of viable MAP were expected in the sample. However, overall, only a few studies utilizing the PMS-PA method for the detection of mycobacteria in real samples have been performed so far. Milk (50 mL individual or BTM) and blood represent the most frequently investigated matrices, and only one publication described the application to fecal samples.

For comparative purposes, other “standard” methods (ELISA, culture, qPCR) were performed in parallel with PMS-PA. A low level of agreement between the results of PMS-PA and culture was recorded for fresh BTM samples, with PMS-PA yielding more MAP positive results than culture. In contrast, when PMS was used instead of the decontamination step before culture on frozen BTM, no significant difference in PMS-PA and PMS-culture positive results was reported, which confirms the adverse effect of decontamination on MAP viability in milk [96]. The moderate agreement between the results of PMS-PA and both PMS-culture and PMS-qPCR for BTM specimens originating from paratuberculosis-affected dairy farms was also documented [43]. However, when individual raw milk samples were tested, poor agreement between PMS-PA and PMS-culture and PMS-qPCR results, respectively, was obtained. The diagnostic specificity and sensitivity of PMS-PA on individual milk samples were assessed to be 100% and 33%, respectively, when the paratuberculosis status of cattle was determined on the basis of fecal culture and serum-ELISA results. These results are superior to those achieved by PMS-culture (96% and 25%, respectively) [97].

One of the main recommendations for how to control paratuberculosis in an affected herd is to feed calf milk replacer (CMR) instead of feeding waste milk to calves. To evaluate if this product is viable MAP-free, a study investigating 83 CMR obtained from dairy farms in the USA was performed by Grant et al. [47]. Using PMS-PA as a detection method, 20.5% of CMR powders showed evidence of a viable MAP presence with numbers of MAP ranging from 6 to 1212 PFU/50 mL of reconstituted CMR. Although it is unknown whether the MAP quantity detected would be sufficient to cause infection in a calf, the possibility of MAP being able to survive the manufacture of dried milk-based products is disquieting, given that these products are destined for consumption by food animals.

It was pointed out earlier that the PMS-PA method is particularly useful for highly complex samples such as feces, which are known to contain components inhibiting phage infection, resulting in no plaque formation. The study by Foddai et al. [96] is so far the only available report where PMS-PA was applied for the rapid and specific detection of viable MAP cells in clinical fecal samples. In this experiment, 51.3% of frozen feces from dairy herds with a known paratuberculosis status gave a positive result by optimized PMS-PA, and it would therefore be interesting to observe PMS-PA performance on fresh fecal samples.

The successful PMS-PA application on cattle blood samples was first reported by Swift et al. [71]. All blood samples originating from paratuberculosis-infected cattle tested positive for MAP presence using optimized PMS-PA and no sample gave a positive result if originating from a certified paratuberculosis-free herd. In this study, no significant difference was observed between PMS-PA employed directly on whole blood samples and the assay performed on the isolated PBMC fraction of blood. Nevertheless, when blood samples from experimentally infected cattle were examined, it was found that the PMS isolation step had a deteriorating effect on assay sensitivity, resulting in very low numbers of plaques produced in MAP-positive samples [93]. Based on these results, PA applied directly on isolated PBMC fractions of blood without the PMS step proved to be the superior approach for the detection of viable MAP in blood. Regardless of these findings, using the PMS-PA method made it possible to detect viable MAP cells in the blood of MAP-exposed animals prior to the onset of clinical signs of disease, suggesting that PA is a suitable means to better understand individual disease stages with respect to the host immune response following MAP exposure.

## 6. Conclusions

In order to control TB in humans, early and accurate diagnosis, followed by prompt and appropriate intervention, is an essential step. PA technology provides detection and enumeration of viable MT cells within 48 h, representing a significant advantage in comparison with conventional culture. However, results of clinical studies have shown that PA has a high specificity but very variable sensitivity in sputum samples. On the other hand, PA is cheaper than PCR, which is particularly important in resource-limited developing countries with a high burden of TB, and its performance characteristics are better compared to smear microscopy. Thus, PA appears to be a valuable cost-effective screening test for TB, allowing timely and appropriate therapeutic decision-making, and a means of monitoring responses to therapy because of its reliance only on the presence of viable mycobacteria.

Regarding veterinary medicine, PA technology has been successfully applied for detection of MAP and *M. bovis* in various matrices with higher sensitivity estimates than conventional culture, suggesting PA is a suitable rapid and sensitive diagnostic tool, which may promote the spread restriction of Johne’s disease and bovine tuberculosis in animal populations and reduction in economic losses in farms due to these diseases.

Despite the satisfactory performance obtained, PA-based methods still remain under development. Original PA requires multiple transfer steps, overnight incubation, and manual extraction of DNA from a plaque, thus the novel adaptation of PA called Actiphage™ was recently developed to overcome these shortcomings providing a more rapid and high-throughput format for the assay. Using this novel assay, the presence of viable MAP and MT complex cells can be detected in human and cattle blood specimens in a substantially shorter time than with original PA, providing a revolutionary tool to study infections caused by the slow-growing mycobacteria.

## Figures and Tables

**Table 1:** Optimization and evaluation of phage amplification assay for the detection of mycobacteria significant in human medicine using artificial contamination of samples.

PA	Bacterial Target	Matrix	Results	Reference
In-house	*M. smegmatis* (2 strains), *M. avium*, *M. bovis* BCG, *M. tuberculosis*	Middlebrook 7H9, LB broth, PBS	At 37 °C, the release of new generation of phages occurred 120 min after infection of *M. smegmatis*. FAS concentration of 10 mM was selected for routine use. D29 phages were able to detect less than 10 CFU of *M. smegmatis*.	[35]
	*M. tuberculosis*	Smear-negative sputum and Middlebrook 7H9	Inhibitory factor of sputum was identified and sample processing method before PA was suggested for its removal. Microcentrifuge-based approach with thixotropic silica was developed. Sodium citrate with calcium chloride offered greater protection (against the virucide) than OADC. Infection time of 1 to 3 h prior to FAS addition was optimal. A liquid culture end-point detection of progeny phages with MTT (colour indicator of viability) was tested—capable of detecting 60 viable MT in 1 mL of sputum.	[55]
	*M. smegmatis, M. bovis* BCG *M. tuberculosis* (14 isolates)	Middlebrook 7H9	To achieve maximum infection rates, inoculation with D29 should not exceed 10^8^ PFU/mL. The length of latent period varies in 13 *M. tuberculosis* isolates. One strain did not support infection by D29. LOD of *M. bovis* BCG was not improved by delaying plating until lysis. Fewer than 10 CFU were detected by PA and samples containing 100 CFU/mL were consistently found to be positive.	[37]
	*M. ulcerans* (18 strains), MT complex (7 strains), *M. avium* (3 strains), *M. scrofulaceum* (2 strains), *M. marinum* (2 strains), *M. fortuitum*, *M. chelonae*, *M. smegmatis*	Middlebrook 7H9 ^1^	Mycobacteriophages D29, L5, Bxz2 and TM4 have the broadest host range. Protocols of PA successfully adapted to *M. ulcerans*. The growth of *M. ulcerans* as well as the phage replication in this host is strongly restricted to temperatures below 35 °C, which can be used for differentiation between *M. ulcerans* and *M. tuberculosis.*	[42]

^1^ Fourteen various mycobacteriophages were used in PA. CFU—colony forming units, LB—Luria–Bertani, FAS—ferric ammonium sulfate, LOD—limit of detection, MT—*Mycobacterium tuberculosis*, OADC—oleate-albumin-dextrose-catalase, PA—phage amplification assay, PBS—phosphate-buffered saline, PFU—plaque forming units.

**Table 2:** Detection of *M. tuberculosis* complex bacteria in human clinical samples.

PA	Matrix (*n*)	Cont. ^+^	TB Treatment	Sensitivity ° (%)	Specificity ° (%)	PPV ° (%)	NPV ° (%)	Other Methods Used	Country	Reference
FPTB	Sputum (1692)	74	NO	75.2 (70.3)	98.7 (99.0)	89 (92)	96 (95)	Smear microscopy, culture	South Africa	[12]
	Sputum (80)	-	*	(27)	(97)	(90)	(55)	Smear microscopy, culture, AMTD	Turkey	[56]
	Sputum (584)	70	n. r.	81.6	97.7	97	85	Smear microscopy, culture	Pakistan	[13]
	Respiratory specimens (sputum, BAL, endotracheal secretion; 50)	-	Various	93.1 /90.6/ (85.3)	88.2 /100/ (100)	93.1 /100/ (100)	88.2 /76.9/ (76.2)	Culture, TB BACTEC culture	India	[52]
	Non-respiratory specimens (pleural fluid, CSF, cold abscess, lymph node, pus, urine; 40)	-	Various	87.5 /90.9/ (59.5)	93.8 /88.8/ (100)	95.5 /90.9/ (100)	83.3 /88.8/ (16.7)			
	Sputum (201)	9	NO	/87.5/	/96.9/	/93.3/	/93.9/	Smear microscopy, culture, TB BACTEC culture, IS*6110* PCR	Turkey	[57]
	Sputum (42)	2	NO	64	93	n. r.	n. r.	Smear microscopy, culture, IS*6110* PCR	Egypt	[14]
	Urine (22)	-	NO	100	100	n. r.	n. r.			
	Sputum (169)	9	NO	77	96	92	87	Smear microscopy, TB BACTEC culture	Pakistan	[58]
	Sputum (193)	78	n. r.	20.7	90.7	42.8	77.2	Smear microscopy, culture	Zambia	[54]
	Sputum (69)	4	n. r.	32	77.5	32	64.6	Smear microscopy, culture, AMTD		
	Respiratory specimens (sputum, BAL; 94) ^1^	-	Various	93.8	83.3	93.8	83.3	Smear microscopy, culture, TB BACTEC culture, AMTD	India	[11]
	Non-respiratory specimens (pleural fluid, urine, tissue, CSF; 45) ^1^	-	Various	95.7	82.4	88	93.3			
	BAL and pleural biopsy (139)	-	NO	(58.8)	(97.9)	(98.1)	(56.5)	Smear microscopy, culture, histopathological examination	India	[59]
	Sputum (200)	-	NO	{76.7}	{86}	n. r.	n. r.	Smear microscopy, culture, BACTEC-MGIT-culture, ESAT-6; 16-23SrRNA PCR	India	[60]
	Smear-negative sputum (208)	101	NO	31.2	94.9	50	88	Culture	Kenya	[7]
	Sputum (68)	-	Various	90.7	96	97.5	85.7	Smear microscopy, culture	India	[8]
	Sputum (196)	-	*	44.3	95.8	87	73	Smear microscopy, culture	South Africa	[61]
FPTB modified	Sputum (196)	-	*	56.7	81.1	68	73			
PhageTek MB	Respiratory specimens (sputum, bronchial, transthoracic or tracheal aspirate, BAL; 2048)	-	Various	|58.3|	|99.1|	|83.2|	|96.9|	Smear microscopy, culture, BacT/ALERT 3D culture	Spain	[5]
	Sputum (206)	2	†	31	86	70	55	Smear microscopy, culture	Philippines	[62]
In-house	Sputum (513)	17	n. r.	44.1	92.4	82.2	67.5	Smear microscopy, culture	Zambia	[37]
	Sputum (115)	5	n. r.	45.3	94.7	88.9	65.1	Smear microscopy, culture, AMTD	Zambia	[54]
	Sputum (1660)	-	n. r.	98.4 (54.8)	71.6 (99)	67.7 (99.6)	98.7 (33.4)	Smear microscopy, culture	China	[63]
	Urine (92) ”	-	n. r.	n. r.	n. r.	n. r.	n. r.	Culture, ELISA, HE staining, immunohistochemistry, immunofluorescence staining	China	[64]
Actiphage	Blood (66)	-	NO	(73.3)	(94–100)	n. r.	n. r.	Smear microscopy, culture, nested PCR, QFT	UK	[53]

^+^ Number of samples excluded from the final evaluation due to overgrowth of contamination on FPTB indicator plates or LJ slopes. * Samples from patients on antituberculosis therapy were excluded from the final analysis. † Some patients on antituberculosis therapy but for less than two weeks duration. ° Sensitivity, specificity, positive and negative predictive values were calculated using conventional culture confirmed diagnosis; automated culture confirmed diagnosis /bound by fraction slash symbol/; conventional and automated culture confirmed diagnosis |bound by absolute value symbols|; PCR confirmed diagnosis {in curly brackets}; or all TB diagnoses (in round brackets). ” The phage amplification assay was performed following culture using colonies grown better on solid culture medium. ^1^ Only 44 respiratory and 40 non-respiratory specimens were tested by PA, as PA was introduced during mid phase of study period. AMTD—amplified *Mycobacterium tuberculosis* direct test, BAL—bronchoalveolar lavage, Cont.—contamination, CSF—cerebrospinal fluid, ESAT-6—early secreted antigentic target of 6 kDa, FPTB—*FASTPlaque*TB™, HE—hematoxylin and eosin, MT—*Mycobacterium tuberculosis*, NPV—negative predictive value, n. r. —not recorded, PA—phage amplification assay, PPV—positive predictive value, QFT—QuantiFERON-TB Gold Plus, qPCR—quantitative polymerase chain reaction, TB—tuberculosis.

**Table 3:** Detection of *M. avium* subsp. *paratuberculosis* and *M. bovis* in real samples by phage amplification assay.

PA	Bacterial Target	Matrix (*n*)	Sample Volume (mL or g)	PA +ve (%)°	Culture +ve (%)	IS*900* qPCR +ve (%)	Sample Origin	Reference
FPTB	MAP	Cattle milk (15)	25	67	-	-	n. r.	[46]
	MAP	Cattle BTM (220) Cattle BTM (225) Sheep BTM (5) Goat BTM (13) Mixed sheep and goat BTM (19) Cheese (28) ^1^	30 30 30 30 30 5/50/15	- 22.2 - - - 0	- 0.9 * 0 0 0 0	28.6 - 20.0 0 5.3 25.0	Cyprus	[69]
	MAP	Cattle BTM (225)	50/30	22.2	0.9 *	-	Cyprus	[48]
	MAP	PIF (32)	1	12.5	9.4 *	21.9	Cyprus	[70]
In-house	*M. bovis*	SCCIT-positive cattle blood (41) SCCIT-negative cattle blood (45)	2 2	66 0	- -	- -	UK	[49]
	MAP	Semi skimmed pasteurized milk (368)	50	10.3	-	-	UK	[71]
Actiphage	*M. bovis*	SCCIT-positive cattle blood (41) SCCIT-negative cattle blood (45)	2 2	95 0	- -	- -	UK	[51]
	MAP	Experimentally infected cattle blood (15) PTB-negative cattle blood (8)	2 2	87 25	93.3 ” 0 ”	- -	UK	[51]

^1^ Four cheeses from cow’s milk, two cheeses from sheep’s milk, one cheese from goat’s milk, eleven cheeses from mixtures of two different milk types and ten cheeses from three different milk types. * Culture-positive samples were confirmed to contain MAP by PCR. ° The identity of mycobacterial cells was confirmed by plaque PCR or RPA. ” Culture was performed from tissue samples. BTM—bulk tank milk, FPTB—*FASTPlaque*TB™, MAP—*Mycobaterium avium* subsp. *paratuberculosis*, n. r.—not recorded, PA—phage amplification assay, PIF—powdered infant formula, PTB—paratuberculosis, SCCIT—Single Comparative Cervical Intradermal Tuberculin.

**Table 4:** Optimization and evaluation of phage amplification assay for the detection of mycobacteria significant in veterinary medicine using artificial contamination of samples.

PA	Bacterial Target	Matrix	Results	Reference
FPTB	MAP (3 strains)	Middlebrook 7H9, UHT whole and semi skimmed milk	In culture medium, MAP kill rate of 2.6–3.3 log_10_ per 1000 mJ/mL UV dose was achieved by UV machine. In whole or semi skimmed milk, MAP kill rate of 0.5–1.0 log_10_ per 1000 mJ/mL UV dose was achieved. PFU counts were consistently 1–2 log_10_ lower than CFU counts.	[72]
	MAP (4 strains), *M. smegmatis*, *M. tuberculosis*, *M. bovis* BCG	Medium FPTB	MAP must be viable to be detected by FPTB. 100% reproducibility, 100% specificity of plaque IS*900* PCR was reached. PCR is not able to reliably amplify a single-copy genomic sequence from the progenitor cell in a plaque. Multiplex plaque PCR for detection *M. bovis* BCG, MT and MAP DNA was established.	[46]
	MAP (2 strains)	UHT whole milk	Significant difference in resistance to UV treatment between two MAP strains was recorded. Inactivation trend was similar for the culture and PA but culture gave consistently higher viable count (of approximately 0.5–2.0 log_10_ units). MAP kill rate of 0.1–0.6 log_10_ was achieved at 1000 mJ/mL regardless of strain used or method employed for MAP enumeration.	[73]
	MAP	Skim milk powder	PA was able to detect MAP in skim milk powder with a LOD of approximately 10 CFU/mL of reconstituted powder.	[70]
FPTB modified	MAP (4 strains)	Medium FPTB, UHT whole and raw milk ^1^	Cell counts by the fluorescence staining method were consistently 1–3 log_10_ lower than CFU/mL and PFU/mL. LOD for FPTB assay: spiked culture medium (6.2 × 10^0^ PFU/mL), UHT milk (1.7 × 10^1^ PFU/mL), raw milk (8.7 × 10^0^ PFU/mL).	[74]
In-house	MAP (5 stains), *M. smegmatis*	Medium FPTB and Middlebrook 7H9, UHT whole milk	The mean burst times for *M. smegmatis* and four MAP strains were 63 and 222 min, respectively. Samples incubated in 2 mM CaCl_2_ overnight before phage addition showed the highest plaque count. The highest number of plaques was found in samples infected with D29 phage for 2 h and incubated for a further 90 min before plating. Plaques were recorded from culture medium and milk samples with less than 10 MAP CFU/mL. For spiked medium and milk, PFU/mL was consistently slightly higher (0.45 log_10_ and 0.23 log_10_, respectively) than CFU/mL.	[68]
	MAP (4 strains)	UHT whole milk	High correlation between CFU/mL and PFU/mL counts obtained for samples before and after thermal stress was observed. PA was able to detect smaller numbers of MAP due to the larger sample volume tested (900 μL) than culture (100 μL). 5.2–6.6 log_10_ reduction in MAP numbers was achieved after heat treatment depending on temperature and time.	[75]
	MAP (3 strains), *M. smegmatis*	Middlebrook 7H9 ^2^	Phage D29 was not able to infect *M. smegmatis* and MAP cells grown under oxygen limiting conditions; on the contrary, phage TM4 could productively infect these cells. Phage D29 is able to infect the mycobacteria more efficiently than TM4 when cells are grown under aerobic conditions. D29 receptors are not altered or lost when mycobacteria are grown under oxygen limiting conditions. The recovery of the productive infectivity of D29, achieved when returning to an aerobic condition, requires *de novo* RNA synthesis.	[67]
	*M. bovis* BCG	Commercial sheep blood	LOD of approximately 10 cells/mL of blood was reached. Efficiency of uptake of *M. bovis* BCG cells by leukocytes of at least 50% was reached. PCR could not consistently detect IS*6110* when only one or two *M. bovis* BCG plaques were present. RPA detected IS*6110* and IS*1081* from samples containing DNA extracted from 5 *M. bovis* BCG plaques, however only RPA IS*6110* primers consistently amplified DNA when agar was extracted from one *M. bovis* BCG plaque mixed with 4 *M. smegmatis* plaques.	[49]
Actiphage	*M. smegmatis*, *M. bovis* BCG, *M. bovis*, MAP (3 strains)	Middlebrook 7H9, commercial sheep blood	Incubation for 180 min should be sufficient to allow D29 to complete its replication cycle in *M. smegmatis*, *M. bovis* BCG and MAP. LOD of Actiphage is lower than that of original PA for MAP and clinical isolate of *M. bovis* in blood. LOD is the same for both Actiphage and original PA when detecting *M. bovis* BCG cells.	[51]

^1^ Raw milk was pooled from three paratuberculosis unsuspicious cows. ^2^ Bacteriophages D29 and TM4 were used in PA. CFU—colony forming units, FPTB—*FASTPlaque*TB™, MAP—*Mycobaterium avium* subsp. *paratuberculosis*, LOD—limit of detection, MT—*Mycobacterium tuberculosis*, PA—phage amplification assay, PFU—plaque forming units, RPA—recombinase polymerase amplification, UHT—ultra-heat treated.

**Table 5:** Optimization and evaluation of peptide-mediated magnetic separation in combination with phage amplification assay for the detection of mycobacteria using artificial contamination of samples.

PA	PMS Type	Bacterial Target	Matrix	Results	Reference
In-house	Manual and automated	MAP (3 strains), *Mycobacterium* sp. ^1^	Middlebrook 7H9, UHT whole milk	MyOne Tosylactivated Dynabeads coated with biotinylated aMp3 and aMptD peptides mixed 50:50 gave 91.5% capture efficiency. The optimized PMS-PA gave average 98.5% capture efficiency and a nonspecific recovery of 5.5%. LOD_50_ of the optimized PMS-PA was 1.9 PFU/mL of culture medium, 7.3 PFU/mL and 14.4 PFU/50 mL of UHT milk. Number of PFU recovered from spiked milk after automated PMS was 10-fold lower than that recovered from spiked culture medium.	[91]
	Automated	MAP (4 strains)	Middlebrook 7H9, raw BTM ^2^, UHT whole milk	The milk pellet is the location of maximal numbers of MAP (>81.5% of initial inoculum) after centrifugation of a milk sample at 2500 *g* for 15 min at ambient temperature. Processing milk on the same day as collection or after overnight storage at 4 °C are the ideal conditions to maximize detection and achieve accurate enumeration of viable MAP cells. Samples can be frozen at −70 °C for up to one month without significant loss of MAP viability. Some evidence of inhibition was obtained for direct qPCR, but not for PMS-qPCR and PMS-PA.	[92]
	Manual	MAP (2 strains)	Middlebrook 7H9, commercial sheep blood	PMS step reduced sensitivity of PA (LOD = 7.3 × 10^2^ PFU/mL of culture media), thus PA directly on isolated PBMC was superior to the PMS step. Detection of MAP directly by PA in PBMC was more reproducible than isolating MAP using PMS on whole blood samples.	[93]
	Automated	MAP (43 strains), *Mycobacterium* sp. ^3^, 5 bacterial isolates ^4^	PBS-T20, UHT whole milk	100% inclusivity of PMS-PA was achieved by testing 43 MAP strains. Of 12 *Mycobacterium* sp. only *Mycobacterium bovis* BCG produced small numbers of plaques. All five bacterial isolates tested negative by PMS-PA. LOD_50_ of the PMS-PA was 0.93 MAP PFU/50 mL UHT milk. PMS-PA proved to be more sensitive than PMS-qPCR and PMS- culture.	[43]
	Manual and automated	MAP (4 strains)	Raw bovine milk ^5^, milk ^6^, skim milk powder	Different sensitivity estimates were obtained for IS*900* qPCR (94%), *F57* qPCR (76%), culture (83%) and PMS-PA (40%) when comparing all matrices. LOD_50_ of PMS-PA was determined to be 3.7 log_10_ CFU/50 mL of both raw and heat-treated milk and 3.2 log_10_ CFU/50 mL of reconstituted milk powder. LOD_95_ of PMS-PA was determined to be 4.3 log_10_ CFU/50 mL of both raw and heat-treated milk and 3.8 log_10_ CFU/50 mL of reconstituted milk powder.	[94]
PA-ELISA in-house	Manual and automated	MAP	Middlebrook 7H9, phosphate buffer, UHT milk, autoclaved bovine faeces	The dynamic range of the assay was found to be approx. 3 × 10^2^–6 × 10^8^ phage/mL. Analytical sensitivity of ELISA was almost identical in phosphate buffer, milk and feces samples. The estimation of MAP number in original specimen is possible by PMS-PA-ELISA. The largest burst size was recorded at high multiplicity of infection. OADC interfered with ELISA and had to be omitted.	[95]
FPTB	Manual	MAP (2 strains)	Commercial horse and sheep blood	Magnetic recovery of beads from blood was found to be inefficient; the centrifugation improved the capture. Horse blood—MAP detection by PMS-FPTB was not successful. Sheep blood—33% of MAP recovered by PMS-FPTB; 1 in 10 dilution of blood resulted in 92% MAP recovery. LOD of PMS-FPTB was determined to be 10 MAP/mL of sheep blood.	[71]

^1^*M. avium* subsp. *avium*, *M. fortuitum, M. kansasii, M. intracellulare, M. bovis* BCG, *M. smegmatis*. ^2^ BTM samples originated from herds without previous history of paratuberculosis. ^3^ *M. avium* subsp. *avium* (2 strains), *M. bovis* BCG, *M. fortuitum*, *M. gordonae*, *M. intracellulare*, *M. kansasii*, *M. marinum*, *M. scrofulaceum*, *M. smegmatis*, *M. terrae*, *M. xenopi*. ^4^ One Gram-positive coccus and four Gram-negative rods. ^5^ Samples collected from four farms tested MAP-negative for at least the last five years. ^6^ Commercial whole pasteurized, UHT whole, semi-skimmed and skimmed milk samples. BTM—bulk tank milk, CFU—colony forming units, FPTB—*FASTPlaque*TB™, LOD—limit of detection, MAP—*Mycobaterium avium* subsp. *paratuberculosis*, OADC—oleate-albumin-dextrose-catalase, PA—phage amplification assay, PBMC—peripheral blood mononuclear cells, PBS-T20—phosphate-buffered saline containing Tween 20, PFU—plaque forming units, PMS—peptide-mediated magnetic separation, UHT—ultra-heat treated.

**Table 6:** Detection of *M. avium* subsp. *paratuberculosis* in real samples by a combination of methods: peptide-mediated magnetic separation and phage amplification assay.

PA	PMS Type	Matrix (*n*)	Sample Volume (mL or g)	PMS-PA +ve (%)°	Culture +ve (%) ”	IS*900* qPCR +ve (%)	Other Method Used	Sample Origin	Reference
In-house	n. r.	Fresh cattle BTM (25) Frozen cattle BTM (19) Frozen bovine faeces (39)	50 50 1	40.0 21.1 51.3	12.0 * (10.5) * -	- - n. r.	-	UK USA USA	[96]
	Manual	Experimentally infected cattle blood (19)	1	36.8	10.5 †	10.5 †	Serum ELISA	Australia	[93]
		JD-negative cattle blood (10)	1	0	0†	0 †			
	Automated	Cattle individual milk (146)	50	21.2	(11.6) *	9.1 ^+,#^	PMS-*F*57 qPCR	UK	[43]
		Cattle BTM (22)	50	59.1	(50.0) *	45.4 ^#^			
		Commercial CMR (83)	1; 5–9	20.5	0; (14.5) *	8.4	-	USA	[47]
		JD-infected cattle individual milk (40)	50	32.5	(25.0) *	-	Serum ELISA, faecal culture	UK	[97]
		JD-free cattle individual milk (105)	50	0	(3.8) *	-			
FPTB	Manual	JD-infected cattle blood (9) JD-free cattle blood (5) JD-different status cattle blood (10) ^1^	1 1 1; 2	100.0 0 80.0	- - (0)	- - -	Serum ELISA, milk ELISA	UK	[71]

^1^ Cows with different milk ELISA results. * Culture-positive samples were confirmed to contain MAP by PCR or whole-genome sequencing. ° The identity of mycobacterial cells was confirmed by plaque PCR. † The method was performed on faecal samples. ” Samples tested positive by conventional culture following a chemical decontamination; or by PMS-culture (in round brackets). ^+^ Only 77 individual raw milk samples were tested by qPCR. ^#^ The IS*900* qPCR followed the PMS isolation step. BTM—bulk tank milk, CMR—calf milk replacer, FPTB—*FASTPlaque*TB™, JD—Johne’s disease, MAP—*Mycobaterium avium* subsp. *paratuberculosis*, n. r.—not recorded, PA—phage amplification assay, PMS—peptide-mediated magnetic separation.

## Data Availability

All data in this article is openly available without any restrictions.
